# Channel Prediction-Based Security Authentication for Artificial Intelligence of Things

**DOI:** 10.3390/s23156711

**Published:** 2023-07-27

**Authors:** Xiaoying Qiu, Jinwei Yu, Wenying Zhuang, Guangda Li, Xuan Sun

**Affiliations:** 1 School of Information and Management, Beijing Information Science & Technology University, Beijing 100192, China; 20182165@bistu.edu.cn (W.Z.); 20172037@bistu.edu.cn (G.L.); sunxuan@bistu.edu.cn (X.S.); 2 School of Information and Communication Engineering, Beijing University of Posts and Telecommunications, Beijing 100876, China; hahayuswag@bupt.cn

**Keywords:** artificial intelligence of things, edge computing, security authentication, intrusion detection

## Abstract

The emerging physical-layer unclonable attribute-aided authentication (PLUA) schemes are capable of outperforming traditional isolated approaches, with the advantage of having reliable fingerprints. However, conventional PLUA methods face new challenges in artificial intelligence of things (AIoT) applications owing to their limited flexibility. These challenges arise from the distributed nature of AIoT devices and the involved information, as well as the requirement for short end-to-end latency. To address these challenges, we propose a security authentication scheme that utilizes intelligent prediction mechanisms to detect spoofing attack. Our approach is based on a dynamic authentication method using long short term memory (LSTM), where the edge computing node observes and predicts the time-varying channel information of access devices to detect clone nodes. Additionally, we introduce a Savitzky–Golay filter-assisted high order cumulant feature extraction model (SGF-HOCM) for preprocessing channel information. By utilizing future channel attributes instead of relying solely on previous channel information, our proposed approach enables authentication decisions. We have conducted extensive experiments in actual industrial environments to validate our prediction-based security strategy, which has achieved an accuracy of 97%.

## 1. Introduction

The combination of core technologies such as 5G, artificial intelligence (AI), and the internet of things (IoT) has opened the door to innovation [1,2]. A new type of IoT structure known as artificial intelligence of things (AIoT) is coming into play. AIoT has become a hot area for realizing real-time information acquisition through IoT sensors and performing intelligent data analysis tasks anywhere along the terminal—edge—cloud continuum. This forms a smart and enabling ecosystem that brings extensive economic benefits [3,4,5]. Benefiting from these advantages, AIoT solutions have expanded into many emerging areas, including commercial surveillance, autonomous driving, smart retail, and drone-based traffic monitoring [6].

AIoT has the potential to offer various new application services [7,8]. AI-based systems have been developed to provide real-time monitoring, analysis, and protection [9,10]. However, to effectively utilize AIoT, networks capable of processing large amounts of information quickly are necessary [11]. Furthermore, the complexity of devices and environments exposes IoT networks to malicious attacks that exploit security vulnerabilities [12]. Due to the large number of IoT sensor nodes and the openness of wireless networks, attackers can eavesdrop on communications, modify transmitted messages, and even send false data [13,14,15]. For instance, in unsupervised industrial IoT networks [16,17,18], clone node attacks can occur, where adversaries hijack control devices and deploy cloned nodes, leading to significant security risks by collecting sensitive information. Industrial control centers may struggle to differentiate these fraudulent nodes, potentially causing serious safety accidents within the AIoT network. In the aforementioned case, the authentication of devices utilizing AIoT applications can be severely compromised, highlighting the increasing concern over the security of AIoT in wireless systems [19].

### 1.1. Existing Methods and Their Challenges

The AIoT network needs to verify the legitimacy of wireless sensors during the initial joining process of communication nodes. The increasing complexity of standard encryption methods has motivated the study of physical layer authentication techniques.Several security technologies have been proposed for IoT networks [13]. For instance, physical unclonable functions (PUF) and wireless fingerprinting (WF) have shown promise in improving authentication in challenging scenarios. Li et al. [20] developed a security framework based on channel virtual representation in millimeter wave (mmWave) massive multiple-input and multiple-output (MIMO) 5G networks, aiming to address a one-class classification problem. Qiu et al. [21] proposed a physical layer authentication framework in IoT networks that utilized a 2D feature measure space for data enhancement. The model’s performance was evaluated using a Gaussian mixture model and tested on the USRP dataset. However, these conventional physical layer approaches are not suitable for future AIoT networks and can be easily compromised by fraudsters, especially in the era of quantum computing.

To enhance authentication in next-generation wireless networks, such as a decentralized, dynamic, and heterogeneous AIoT network, researchers have explored the concept of lightweight flexible group authentication mechanisms for fingerprint identification [22,23,24,25]. A group authentication scheme was proposed in [23,24] to detect devices’ identities based on generated tokens for decentralized edge collaboration. Additionally, a game theory framework was proposed to extract random characteristics of IoT devices, enabling the cloud to effectively verify signal reliability [26]. A hybrid privacy-preserving mechanism for the IoT is introduced in [27], employing the federated learning (FL) method to identify malicious participants. Gao et al. [28,29] conducted research on the impact of PUF-based deep learning in wireless sensor networks, specifically focusing intelligent spoofing. They compared the results of several adversarial attacks with deep Q networks. Wang et al. [30,31] developed a novel CSCB fingerprinting framework to detect spoofing attacks. Their proposed scheme utilizes sector-level sweep (SLS) trace-based fingerprinting to enhance effectiveness in mmWave 60-GHz IEEE 802.11 ad networks. Furthermore, the authors in [32] developed a graph neural network (GNN) to effectively detect message injection in control area networks. Other deep learning (DNN)-based security authentication methods are also mentioned in the literature [33,34]. However, the authentication approaches of [25,32] remain inflexible and risk-agnostic in future AIoT network deployments and have low authentication reliability. These solutions also exhibit low authentication reliability and fail to address robustness improvements in dynamic environments. Additionally, the PUF algorithms introduced in [13], do not fully account for changes in the surrounding environment or the time-varying properties of the channel. In a nutshell, a new learning-based dynamic authentication solution is highly beneficial for the next generation of IoT networks. Such a technique should encompass a comprehensive physical layer security scheme that allows IoT devices to authenticate without sharing keys.

### 1.2. Contributions

This paper proposes a novel dynamic authentication scheme that leverages an intelligent learning model capable of predicting future channel features. In future AIoT networks, the cloud may be unable to identify all transmission signals from access sensor nodes due to limited computing resources and network heterogeneity. Therefore, in a real wireless communication system, the control center must perform dynamic intelligent authentication for a large number of IoT devices. The main objective of this research is to present an intelligent framework that integrates new ideas from dynamic feature extraction and prediction to achieve computationally-efficient authentication of smart nodes.

The key contributions of this paper can be summarized as follows:

- A Savitzky–Golay filter (SGF) is utilized to preprocess wireless channel estimation, aiming to improve spectrum smoothness and reduce interference. Then, the relationship between time series and dynamic characteristics of wireless channels is exploited to extract fingerprints of IoT devices using the high order cumulant model (HOCM). This SGF-HOCM feature extraction enables the edge computing node to effectively track the channel model during two adjacent communications;
- An intelligent framework is proposed to enable the receiver to verify the reliability of received signals and detect the presence of network fraudsters attempting to compromise security performance. The proposed deep learning scheme employs long short-term memory (LSTM) blocks to predict dynamic fluctuations in channel information elements. This allows the security framework to effectively utilize predicted channel information for authentication instead of relying solely on previously estimated data;
- Simulations are conducted using open datasets from the National Institute of Standards and Technology (NIST). The results demonstrate that the proposed learning algorithms enhance the authentication performance of the system. This improvement makes the method highly valuable for time-varying channel prediction, dynamic feature extraction, and security authentication.

The remainder of this article is organized as follows: the system model and analysis are introduced in Section 2. The proposed authentication scheme is described in detail in Section 3, followed by simulation and experimental verification for our dynamic authentication strategy in Section 4. Finally, the paper concludes in Section 5.

## 2. System Model

**Figure 1:**
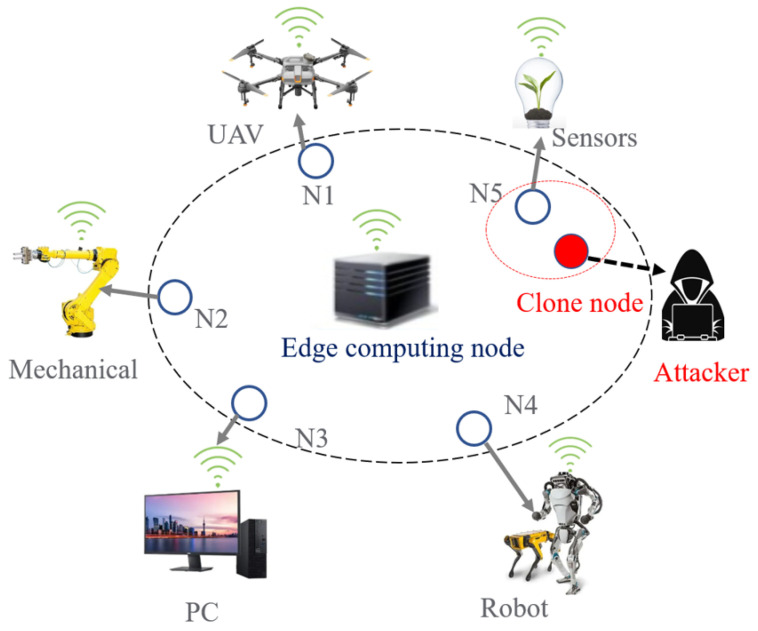
High-level architecture and system model. N1, N2, *…*, and N5 are all legitimate IoT devices. The attacker replicated the clone node based on a legitimate node, such as N5.

We introduce a clone attack scenario, as shown in Figure 1. The legitimate receiver is the edge computing node, which intends to communicate with other IoT devices, including N1, N2, *…*, and N5. An attacker imitates the identity of legitimate transmitter N5 and creates a clone node that injects illegal messages to the edge computing node. The clone node participates in data communication with industrial edge computing. The edge computing node needs to authenticate messages to detect whether they are from legitimate wireless devices.

The extraction of the physical layer channel response is performed by the legitimate receiver. According to the wireless channel model [35,36], the expression of the received signal can be written as

(1) y(t)=h(t)∗x(t)+n(t)

where *t* is the time slot, *h* denotes the channel impulse response, *x* is a pilot signal known to the transmitter and receiver for estimating channel information, and *n*(*t*) is the additive white Gaussian noise with variance $\sigma^{2}$. The corresponding frequency-domain representation obtained through Fourier transform is

(2) $$Y\left(f_{k},t\right)=H\left(f_{k},t\right)X\left(f_{k}\right)+N\left(f_{k},t\right)$$

where *Y*, *H*, *X*, and *N* represents *y*, *h*, *x* and *n*, respectively, in frequency domain. $f_{k}$ is the frequency of the *k*th subcarrier. Then, the wireless channel estimation can be given by

(3) $$\hat{H}\left(f_{k},t\right)=\frac{Y(f_{k},t)}{X(f_{k})}=H\left(f_{k},t\right)+\hat{N}\left(f_{k},t\right)$$

where

(4) $$\hat{N}\left(f_{k},t\right)=\frac{N(f_{k},t)}{X(f_{k})}.$$

From the wireless channel model in (3), we have the channel estimations of different receivers as

(5) $${\hat{H}}_{a}\left(f_{k},t\right)=H_{a}\left(f_{k},t\right)+{\hat{N}}_{a}\left(f_{k},t\right)$$

(6) $${\hat{H}}_{c}\left(f_{k},t\right)=H_{c}\left(f_{k},t\right)+{\hat{N}}_{c}\left(f_{k},t\right).$$

where ${\hat{N}}_{a}\left(f_{k},t\right)$ and ${\hat{N}}_{c}\left(f_{k},t\right)$ in (5) and (6) are the channel estimation errors, and *a* and *c*, respectively, denote legitimate node A and clone node C. Different positions of the wireless device indicate different channel characteristics. Therefore, the channel estimations of the legitimate node are supposed to be different from that of the cloned node, that is

(7) $${\hat{H}}_{a}\left(f_{k},t\right)\neq {\hat{H}}_{c}\left(f_{k},t\right).$$

We first analyze the traditional problem of binary hypothesis testing. The authentication can be formulated as

(8) $$\left\{\begin{array}{c} H_{0}:{\hat{H}}_{i}\left(t+1\right)\to {\hat{H}}_{a}\left(t\right),\\ H_{1}:{\hat{H}}_{i}\left(t+1\right)\to {\hat{H}}_{c}\left(t\right), \end{array}\right.$$

where $H_{0}$ indicates that the future estimation ${\hat{H}}_{i}\left(t+1\right)$ is an authentic packet from legitimate device A, and $H_{1}$ means that ${\hat{H}}_{i}\left(t+1\right)$ comes from different wireless transmission terminals, such as a cloned node.

Existing methods compare the channel measurements received at adjacent times within the channel coherence time, and then determine whether the variables are from a legitimate sender or a malicious attacker, just like the authentication problem in (8). We have adopted an authentication classification function based on machine learning, without using the attacker’s channel information, which can be described as

(9) $$\left\{\begin{array}{c} H_{0}:f\left({\hat{H}}_{a}\left(t\right),{\hat{H}}_{i}\left(t+1\right)\right)<\eta ,\\ H_{1}:f\left({\hat{H}}_{a}\left(t\right),{\hat{H}}_{i}\left(t+1\right)\right)\geq \eta , \end{array}\right.$$

where f(·) is a function that quantifies the difference between the previous value ${\hat{H}}_{a}\left(t\right)$ and future estimation ${\hat{H}}_{i}\left(t+1\right)$, η denotes an attack threshold. In this paper, we directly use the estimated channel matrices $\hat{H}$, and then consider a physical layer authentication strategy to detect malicious attacks. There are several algorithms to obtain wireless channel estimations [37,38,39,40,41].

## 3. Intelligent Prediction-Based Authentication Strategy

**Figure 2:**
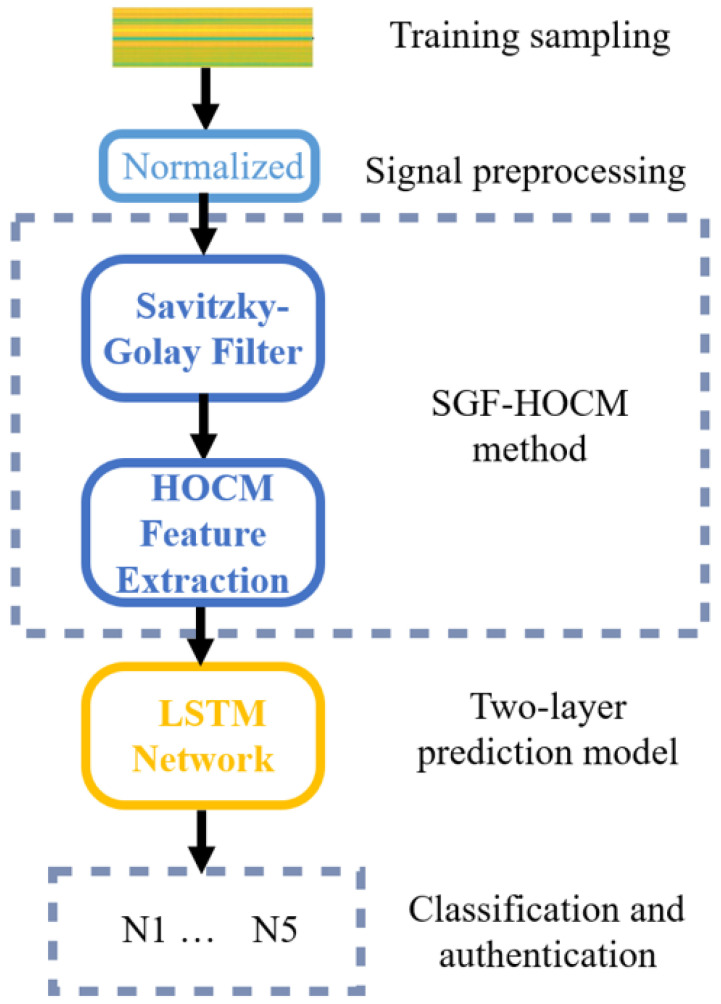
The proposed model of security authentication.

The proposed authentication strategy based on intelligent prediction consists of four components, as shown in Figure 2. The security model uses physical layer attributes to prevent cloning attacks. The wireless characteristics are learned using the SGF-HOCM method. This derives time-varying features from preprocessed data using Savitzky–Golay filtering and HOCM feature extraction. Using the extracted features as input, two-layer LSTM network is trained to predict time-varying channel parameters. Finally, the predicted values are compared with the actual values to identify different IoT nodes.

### 3.1. Channel Information Processing Based on SGF-HOCM

The channel measurement value is vulnerable to the interference of channel estimation error and environmental noise. In view of this analysis, it can be concluded that Gaussian noise and estimation error in (1) and (3) are the main factors to impair the authentication model. These urge us to explore an effective authentication scheme based on time-varying channel prediction to improve the robustness and reliability of the authenticator.

SGF is widely used in data stream smoothing and denoising, and is a filtering method based on local polynomial least square fitting in the time domain. The biggest advantage of SGF is that it can ensure that the shape and width of the signal remain unchanged while filtering out noise. The filtering effect of SGF varies with the selected window width, which can meet the needs of various occasions. The mathematical expression of SGF is formulated as follows:

(10) $$h_{k,smooth}=\frac{1}{2w+1}\sum_{i=-w}^{+w}h_{k+i}$$

where *w* is the length of the window and *k* denotes the order of the polynominal. The smaller the value of *w*, the closer the curve is to the actual curve. The *k* value is also important for smoothing curves. The larger the *k* value, the closer the curve is to the real curve, whereas the smaller the *k* value, the smoother the curve is. In addition, when the value of *k* is large, due to the limitation of the window length, fitting may encounter problems, such as high-frequency curves becoming straight lines.

Due to the time-varying nature of wireless links and the difficulty of tracking changes, the existing methods have limited authentication capabilities for intelligent access terminals. One of the main advantages of HOCM is that it contains both amplitude and phase information [42]. Therefore, HOCM is very likely to be a matrix in the authentication scheme, providing a robust feature extraction method. As previously mentioned, a key technology for enabling intelligent prediction models for clone node detection in wireless networks is to extract key features. Assuming that $\left\{x_{1}^{\prime },x_{2}^{\prime },\cdots ,x_{d}^{\prime }\right\}$ is the channel estimations after SGF, their corresponding *d*th-order cumulant can be defined as the coefficient of $\left\{v_{1},v_{2},\cdots ,v_{d}\right\}$ in the Taylor series expansion of the cumulant-generating function

(11) $$\psi \left(v\right)=InE\{exp\left(jvx^{\prime }\right)\}$$

where E[·] is a mathematical expectation operator, representing the statistics average. The *d*th-order cumulant of $x^{\prime }$ is defined as

(12) $$cum\left(x_{1}^{\prime },x_{2}^{\prime },\cdots ,x_{d}^{\prime }\right)={\left(-j\right)}^{d}\left[\partial /\partial v_{1}\partial v_{2}\cdots \partial v_{d}\right]\psi \left(v\right){|}_{v=0}.$$

Because the mathematical expressions of the third order and above are very complicated, zero-average processing is used for the channel estimates in the practical application of the security authentication, to simplify the high-order cumulant. When the random variable $\left\{x^{\prime }\left(t\right)\right\}$ is a zero mean, the *d*th-order cumulant is defined as

(13) $$\begin{array}{cc} C_{kk} & =(\Delta_{1},\Delta_{2},\cdots ,\Delta_{d-1})\\ & =cum(x^{\prime }\left(t\right),x^{\prime }\left(t+\Delta_{1}\right),\cdots ,x^{\prime }\left(t+\Delta_{d-1}\right)) \end{array}$$

where $\Delta_{1},\Delta_{2},\cdots ,\Delta_{d-1}$ are the time delays.

According to (12) and (13), the mathematical expressions of the corresponding second moment, third moment and fourth moment of $x^{\prime }\left(t\right)$ are then formulated as follows [42]:

(14) $$C_{2x^{\prime }}\left(\Delta \right)=E\left\{x^{\prime }\left(t\right)x^{\prime }\left(t+\Delta \right)\right\}$$

(15) $$C_{3x^{\prime }}\left(\Delta_{1},\Delta_{2}\right)=E\left\{x^{\prime }\left(t\right)x^{\prime }\left(t+\Delta_{1}\right)x^{\prime }\left(t+\Delta_{2}\right)\right\}$$

(16) $$\begin{array}{cc} C_{4x^{\prime }}\left(\Delta_{1},\Delta_{2},\Delta_{3}\right)= & E\{x^{\prime }\left(t\right)x^{\prime }\left(t+\Delta_{1}\right)x^{\prime }\left(t+\Delta_{2}\right)x^{\prime }\left(t+\Delta_{3}\right)\}\\ \; & -C_{2x^{\prime }}\left(\Delta_{1}\right)C_{2x^{\prime }}\left(\Delta_{2}-\Delta_{3}\right)-C_{2x^{\prime }}\left(\Delta_{2}\right)C_{2x^{\prime }}\left(\Delta_{3}-\Delta_{1}\right)\\ & -C_{2x^{\prime }}\left(\Delta_{3}\right)C_{2x^{\prime }}\left(\Delta_{1}-\Delta_{2}\right) \end{array}$$

In this paper, the SGF-HOCM analysis method is introduced for signal processing of wireless channel information.

### 3.2. Channel Prediction Based on Two-Layer LSTM

**Figure 3:**
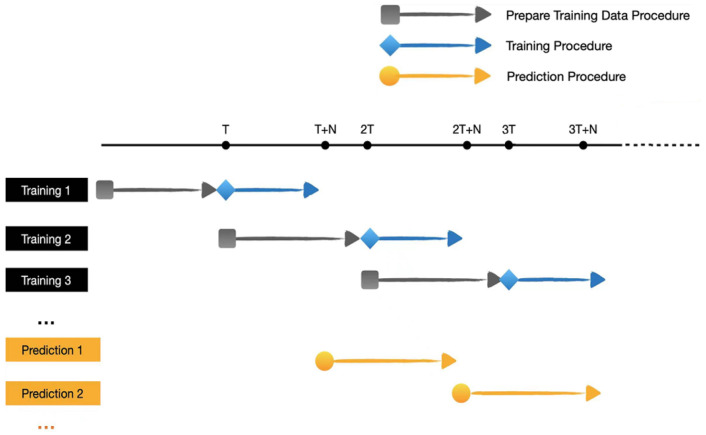
The timing schedule for training and prediction.

The channel estimations processed by SGF-HOCM method form a sequence, which serves as the input of the two-layer LSTM network. Let the previously SGF-HOCM preprocessed finite segment be the training dataset of two-layer LSTM model, shown as $H_{train}=\left[h_{p}^{\prime \prime },h_{p-1}^{\prime \prime },h_{p-1}^{\prime \prime },\cdots ,h_{1}^{\prime \prime }\right]$, where *p* is the size of LSTM training data. The original data of the testing sample is shown as $H_{test}=\left[h_{p+1}^{\prime \prime },h_{p+2}^{\prime \prime },h_{p+3}^{\prime \prime },\cdots ,h_{p+q}^{\prime \prime }\right]$, where *q* denotes the size of LSTM testing data. Specifically, we consider a model with ten inputs to predict channel vector in the future, as ${\tilde{h}}_{p+1}$. The prediction procedure can be expressed as

(17) $$\begin{array}{cc} {\tilde{h}}_{p+1} & =L(h_{p}^{\prime \prime },h_{p-1}^{\prime \prime },h_{p-2}^{\prime \prime },\cdots ,h_{p-9}^{\prime \prime }),\\ {\tilde{h}}_{p+2} & =L(h_{p+1}^{\prime \prime },h_{p}^{\prime \prime },h_{p-1}^{\prime \prime },\cdots ,h_{p-8}^{\prime \prime }),\\ {\tilde{h}}_{p+3} & =L(h_{p+2}^{\prime \prime },h_{p+1}^{\prime \prime },h_{p}^{\prime \prime },\cdots ,h_{p-7}^{\prime \prime }),\\ \vdots & \;\\ {\tilde{h}}_{p+q} & =L(h_{p+q-1}^{\prime \prime },h_{p+q-2}^{\prime \prime },h_{p+q-3}^{\prime \prime },\cdots ,h_{p+q-10}^{\prime \prime }) \end{array}$$

where L(·) is the prediction function of LSTM model. In our two-layer LSTM network, the predictor always uses the original data in the training step. For instance, we predict ${\tilde{h}}_{p+q+1}$ based on the same function, whereas the inputs are updated to $h_{p+q}^{\prime \prime },h_{p+q-1}^{\prime \prime },h_{p+q-2}^{\prime \prime },\cdots$, $h_{p+q-9}^{\prime \prime }$. The timing schedule for training and prediction is shown in Figure 3.

One of the attractions of a predictor is that it can use previous channel information to predict future channel attributes (i.e., ${\tilde{H}}_{a}\left(t+1\right)=\left[{\tilde{h}}_{1},{\tilde{h}}_{2},\cdots ,{\tilde{h}}_{L}\right]$) of legitimate node. Mathematically, the parameters of two-layer LSTM can be formulated as [43,44]

(18) $$f^{t}=\sigma \left(W^{f}\cdot \left[h^{t-1},x^{t}\right]+b^{f}\right)$$

(19) $$f_{t}=\sigma \left(W_{f}x_{f}+R_{f}h_{t-1}+b_{f}\right)$$

(20) $$i_{t}=\sigma \left(W_{i}x_{t}+R_{i}h_{t-1}+b_{i}\right)$$

(21) $$o_{t}=\sigma \left(W_{o}x_{t}+R_{o}h_{t-1}+b_{o}\right)$$

(22) $${\tilde{c}}_{t}=\tanh\left(W_{c}x_{t}+R_{c}h_{t-1}+b_{c}\right)$$

(23) $$c_{t}=f_{t}*c_{t-1}+i_{t}*{\tilde{c}}_{t}$$

(24) $$h_{t}=o_{t}*\tanh c_{t}$$

where $f^{t}=0$ represents complete blocking of information, $f^{t}=1$ denotes passing information, and the notations are defined in Table 1.

In this paper, the mean squared error (MSE) is the loss function in the predictor network. MSE is popular as a measure because it is sensitive to outliers and provides greater penalties [43]. MSE can be formulated as

(25) $$MSE=\frac{1}{L}\sum_{i=1}^{L}e_{i}$$

(26) $$e_{i}=\frac{1}{Q_{1}Q_{2}}\sum_{Q_{1},Q_{2}}{\left(H_{Q_{1},Q_{2}}^{\prime \prime }-{\tilde{H}}_{Q_{1},Q_{2}}\right)}^{2}$$

where *L* represents the number of channel samples, *e_i_* is the $Q_{1}\times Q_{2}$ element-wise mean squared error, and $H^{\prime \prime }$ and $\tilde{H}$ denote the real measurement after SGF-HOCM processing and the predicted value of LSTM network, respectively. Wireless channel prediction is achieved by first SGF-HOCM processing an estimation sequence ${\hat{H}}_{a}$, and then forecasting the future channel value ${\tilde{H}}_{a}\left(t+1\right)$.

Through the above two-layer LSTM predictor, we aim to track time-variant channel values. The parameters of the prediction network model are summarized in Table 2. In other words, we can directly use the observed channel estimation and the prediction values to perform the authentication in Section 3.3.

### 3.3. Prediction-Based Authentication Model

Once we obtain the predicted value ${\tilde{H}}_{a}\left(t+1\right)$ at time t+1, we will perform physical layer authentication. The proposed scheme constructs the authentication process based on the predicted channel information of legitimate nodes. The authentication problem in (9) is reconstructed as

(27) $$\left\{\begin{array}{c} H_{0}:MSE\left({\tilde{H}}_{a}\left(t+1\right),H_{i}^{\prime \prime }\left(t+1\right)\right)<\eta ,\\ H_{1}:MSE\left({\tilde{H}}_{a}\left(t+1\right),H_{i}^{\prime \prime }\left(t+1\right)\right)\geq \eta , \end{array}\right.$$

where ${\tilde{H}}_{a}\left(t+1\right)$ represents the predicted future characteristics of legitimate node A, and $H_{i}^{\prime \prime }\left(t+1\right)$ is the real observation. Since the wireless channel attributes are dynamic, we compare the predicted channel features with the real observations of time t+1, instead of comparing the values (i.e., $H_{a}^{\prime \prime }\left(t\right)$ and $H_{i}^{\prime \prime }\left(t+1\right)$) of two adjacent times to make authentication decisions. The MSE between the predicted value and the actual value is used as a metric. According to the information in (27), we obtain the acceptance region of legitimate node A. If the MSE between the predicted channel characteristics and the observed samples is greater than the threshold *η*, the transmission should be denied.

To evaluate the prediction and authentication results, two performance metrics (i.e., $R^{2}$ and Loss value) are used to measure the accuracy of the dynamic authentication model. For a prediction-based authenticator, higher $R^{2}$ means better authentication capability. For instance, $R^{2}=1$ indicates that the predicted data exactly matches the actual data. The predictor we trained perfectly predicts all the real time-varying information. If $R^{2}=0$, that is, each predicted value of the sample is equal to the mean value, then the trained authentication model has poor accuracy. The formula of $R^{2}$ can be expressed as

(28) $$R^{2}=1-\frac{MSE}{Var}$$

where Var is the variance and MSE is the mean squared error in (25). To sum up, we introduce the SGF-HOCM processing method and the authenticator based on LSTM prediction. In the dynamic learning model, we test a variety of combinations of processing steps to find the best-performing authenticator with LSTM prediction method. The results of dynamic authentication scheme are reported in the next section.

## 4. Results and Discussions

### 4.1. Measurement Setup

**Figure 4:**
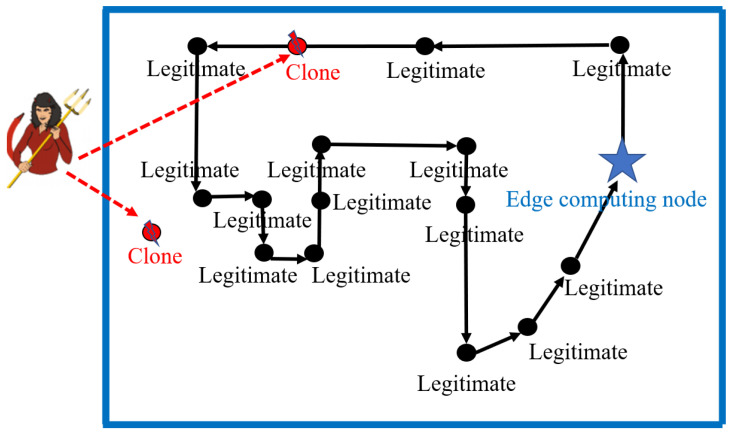
Simulation experiment under automotive assembly using NIST datasets. Fix the transmitter while moving the receiver at a constant rate to measure at continuous points throughout the entire facility.

In this section, we use the channel information dataset provided by NIST in the automotive factory to simulate malicious attack scenarios. As shown in Figure 4, a typical multi-acre transmission assembly factory of the automotive industry is selected for radio frequency propagation measurement [45]. The floor size of the automotive factory is more than 400 m × 400 m. The ceiling is about 12 m high. In this scenario, a channel sounder system is used to take the measurements at a continuum of points throughout the facility by fixing the transmitter and moving the receiver at a constant rate. The analysis is based on channel impulse response data collected using equipment developed by NIST. The NIST channel sounder measurement system is a positive-negative sequence correlation system, which consists of a single sender with a power amplifier and a receiver [45]. The transmitter continuously transmits a sequence of positive–negative digital symbols modulated by a binary phased-shift keying signal, and is up-converted to a radio frequency carrier frequency. After passing through the power amplifier, the signal traverses the automotive factory and is detected by the channel sounder receiver. The statistics of channel estimates include frequency, expected value of the path loss exponent, delay, delay spread, and K-factor. The frequency is 5.4 GHz, the expected path loss exponent is 3.6, the delay is 644.4 ns, the delay spread is 177.4 ns, and the K-factor is 4.7 dB. The dataset splits into two sets: training (60,000 packets) and testing (2000 packets). From the training set, 10% of randomly selected samples are put aside and used for validation.

### 4.2. Performance of Feature Extraction

**Figure 5:**
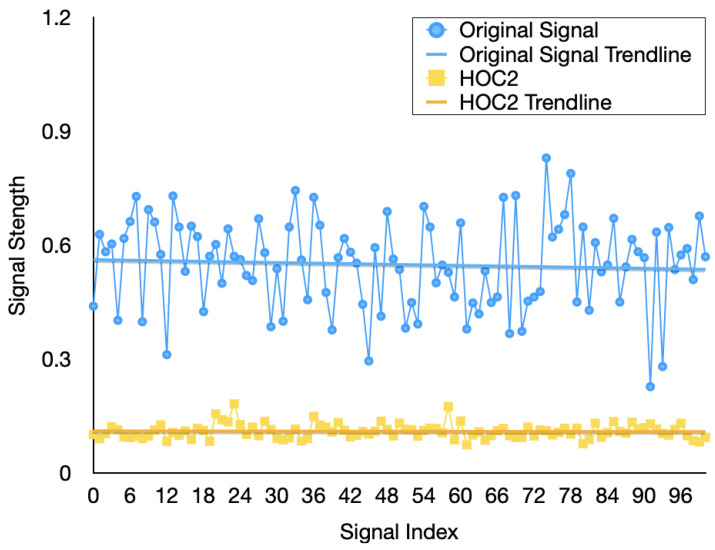
Extracted channel features using the different HOC2 methods.

**Figure 6:**
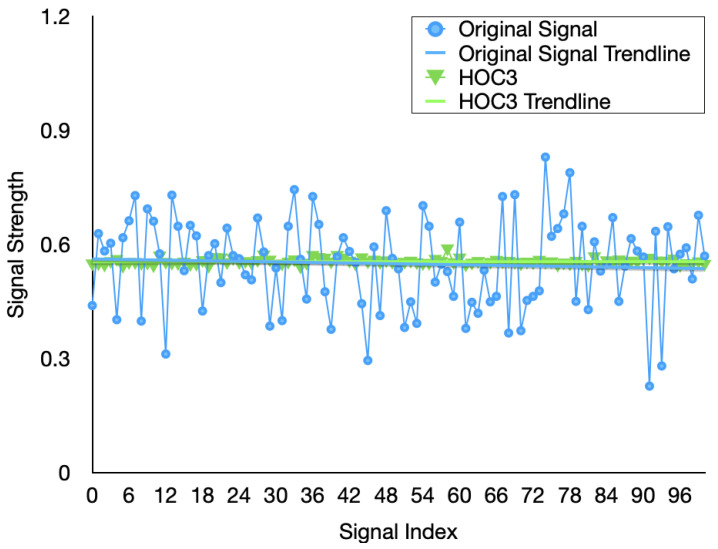
Extracted channel features using the different HOC3 methods.

**Figure 7:**
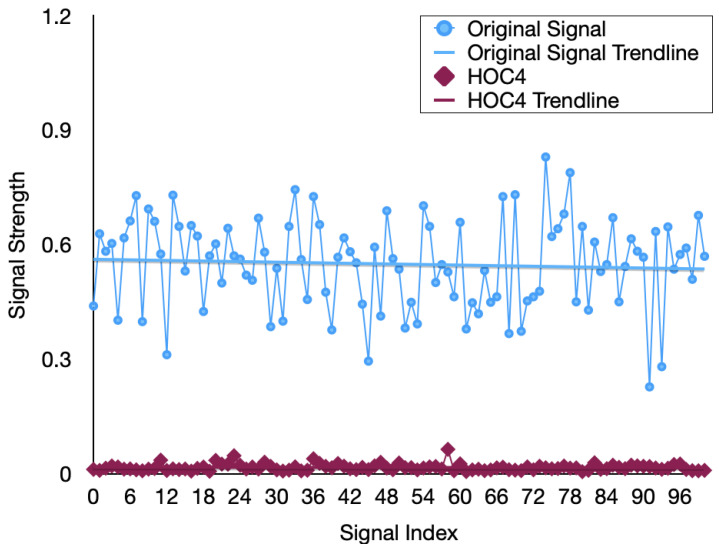
Extracted channel features using the different HOC4 methods.

In order to achieve denoising and feature extraction, we propose the SGF-HOCM method to process the channel estimates. This section first determines the optimal order cumulant to obtain the SGF-HOCM processing process. In the following section, we evaluate the effectiveness of using the proposed HOC3-based approach. The previously estimated channel data is divided into training and testing, in which 58,000 training samples are used for training the predictor, while 2000 testing samples are used for verification. The features extracted from different order domains are demonstrated in Figure 5, Figure 6 and Figure 7. The simulation results certify the effectiveness of our HOC3 strategy. Because the third-order cumulant of channel estimation is superior to the second-order and fourth-order cumulants, the third-order cumulant is selected. The HOC3 preprocessing signal matches the measurement very well. The advantage of the HOC3 method in denoising is that it shows more promising performance, while the improvement in HOC2 and HOC4 methods is limited. We can observe that the SGF-HOCM step can extract useful features and minimize the impact of noise. According to the above description, we can reasonably select an optimal order cumulant for subsequent prediction during feature extraction process of time-varying channels. We utilize SGF-HOCM to preprocess the estimated values and provide training sample for the prediction model.

### 4.3. Comparison of Prediction Performance

**Figure 8:**
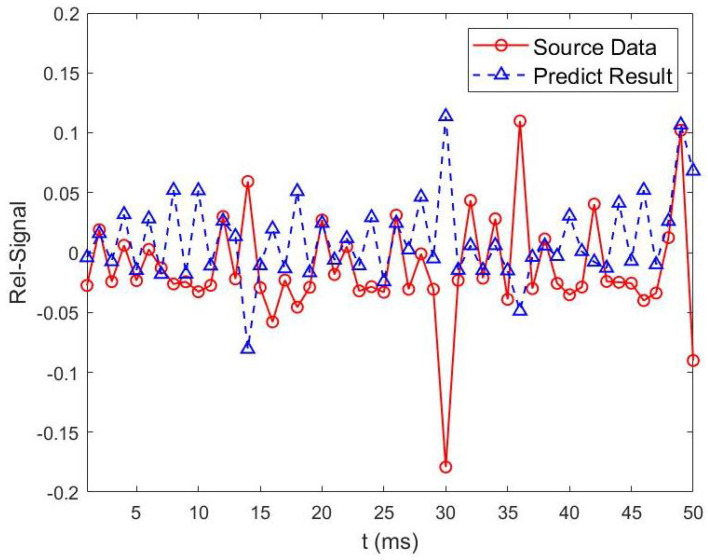
No preprocessing method is used for channel information prediction based on two-layer LSTM scheme.

**Figure 9:**
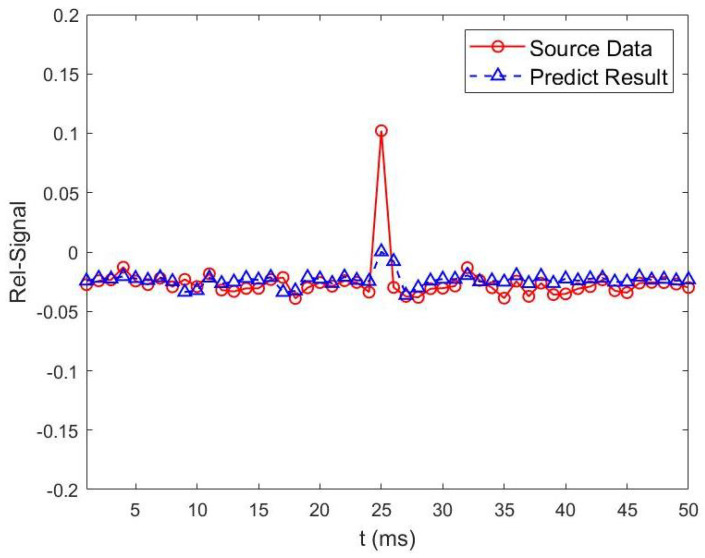
SGF-HOCM method is used for channel information prediction based on two-layer LSTM scheme.

As shown in Figure 8 and Figure 9, to achieve accurate prediction of wireless channel information, two different preprocessing methods are compared. From Figure 8 and Figure 9, we can see that, the predicted future values based on our proposed SGF-HOCM-assisted LSTM scheme match the real channel estimates very well. As described above, the denoising and dynamic feature extraction of channel sequences are important factors affecting the accuracy of the predictor. The performance of the prediction-based authenticator depends on the features of channel estimation, and the LSTM learning model could perform better with high complexity and strong time-varying series. Therefore, we introduced the SGF-HOCM processing method in dynamic authentication strategies to ensure the superiority of denoising.

### 4.4. Comparison of $R^{2}$ Performance

**Figure 10:**
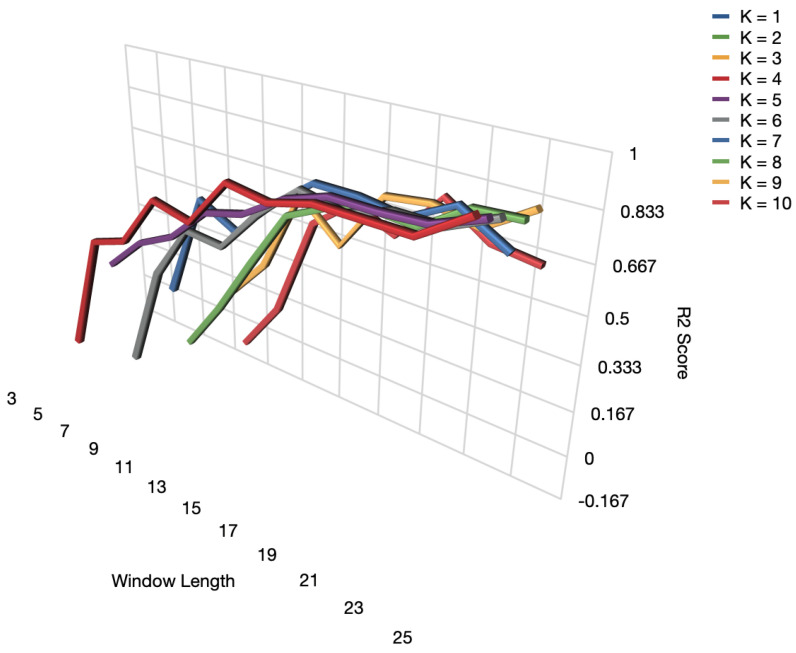
Performance of $R^{2}$ under different SGF parameters.

We compared the security performance of the predictor where $R^{2}$ has been applied for the authentication function. Figure 10 shows the $R^{2}$ curve of dynamic forecasting model. $R^{2}>0.8$ shows the forecasting performance, which is desirable for malicious node identification in wireless networks. We further discussed the potential reasons for using forth order polynominal in SGF-HOCM-assisted LSTM scheme.

### 4.5. Training Performance

**Figure 11:**
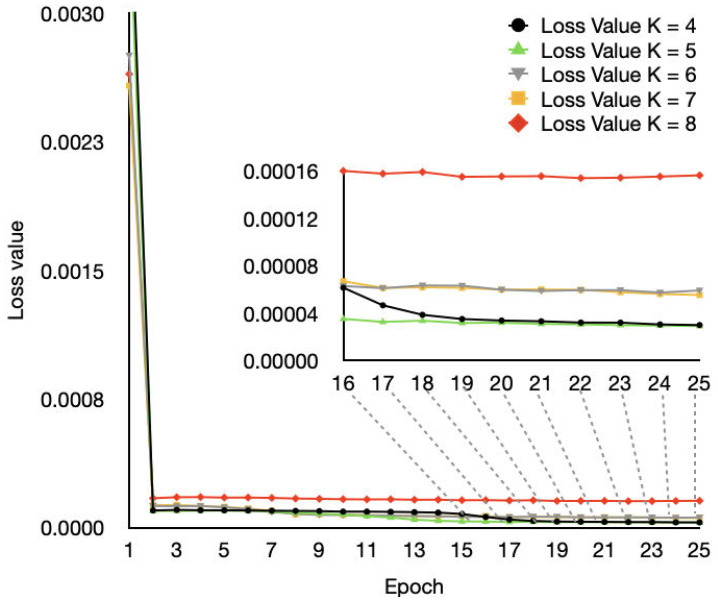
Training performance of the proposed SGF-HOCM-assisted LSTM scheme.

To capture the training performance of our proposed SGF-HOCM combined with LSTM approach, we provide the loss value of the network, as shown in Figure 11. We considered a two-layer LSTM network on the cloud, which is very useful in channel information prediction. From Figure 10, we know that *k* = 4 results in higher authentication performance. We can see from Figure 11 that the loss values of LSTM is approximately zero after the number of iterations is greater than 15. Note that when the proposed scheme can accurately predict future channel information, the verifier can compare the predicted values with the next actual observation results to achieve malicious attacker detection.

### 4.6. Authentication Performance

**Figure 12:**
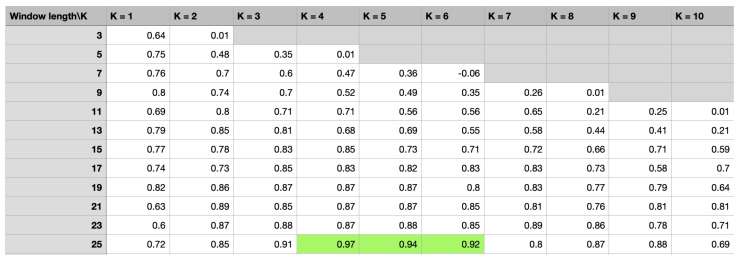
$R^{2}$ authentication performance of the proposed scheme.

In addition, Figure 12 shows the impact of two important parameters in SGF on $R^{2}$, namely, the length of the window *w* and a *k*th order polynominal. From the table, we observe that when k=4, $R^{2}$ achieves the best performance, which is $R^{2}$ = 0.97. The results represented by the green line have achieved high predictive performance. The SGF-HOCM-assisted LSTM scheme does not require key transmission, which avoids problems with possible key leakage. In addition, we note that increasing the number of layer and window length increases both accuracy and computational time overhead. Therefore, as shown in Figure 12, the proposed method uses a two-layer LSTM network with a window length of 25 to balance computation time and authentication performance. More importantly, physical layer security authentication does not depend on computational complexity and can accurately quantify security. By contrast, the key-based cryptography approach requires more time and complexity, which is problematic for sensor devices. Thus, given the potential of LSTM for PLUA in AIoT, dynamic authentication mechanisms have considerable interest in future IoT systems.

**Figure 13:**
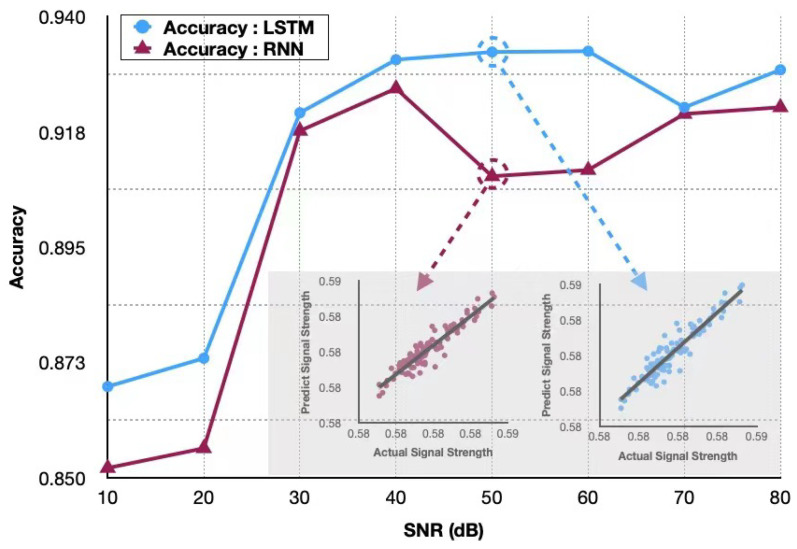
Authentication accuracy comparison between proposed LSTM scheme and the traditional RNN scheme.

We demonstrate the superiority of our proposed SFG-HOCM-assisted LSTM scheme by comparing with the traditional RNN scheme, which only exploited HOC to model the time-varying channel. Figure 13 shows a clear comparison of accuracy between LSTM method and RNN approach under different signal-to-noise ratios. Our SGF-HOCM-assisted LSTM scheme shows a more promising performance due to the superiority of LSTM in predicting future channel characteristics, and it has a significant improvement when exploiting SGF-HOCM preprocessing, while the traditional method only shows limited promotion.

## 5. Conclusions

In this work, we have developed a dynamic authentication mechanism to address the security challenges in next-generation AIoT networks. We adopted SGF-HOCM processing method to extract time-varying characteristics based on physical layer attributes. We used a two-layer LSTM algorithm to predict future channel vectors based on existing channel information, and compared them with observed channel variations extracted from the transmitter to perform security detection. We proposed an intelligent authentication scheme, which only needs the channel information of legitimate nodes, and avoids using the channel model of spoofing devices. Finally, we conducted a simulation using the dataset from the National Institute of Standards and Technology, demonstrating the advantages of the proposed dynamic authentication scheme.

This channel prediction-based security authentication scheme was shown to achieve a very high accuracy compared to other methods. Although the maximum accuracy is high, $R^{2}$ = 0.97, there is room for future work. One is in the area of preprocessing engineering and feature selection with the goal of creating better prediction-based models. Although the SGF-HOCM feature vector that is based on Savitzky–Golay filter and HOCM method have been used successfully, other attribute characteristics are possible, and the use of more than two filters could be considered. The training efficiency of LSTM is much lower than that of traditional RNN under the same computational power. LSTM alleviates the long-term dependency problem of RNN, but for longer sequence data, it requires higher computational complexity and longer training time. Another important study would be to implement this security algorithm in a real AIoT system in order to evaluate its performance under real conditions and in different scenarios.

## Figures and Tables

**Table 1:** Parameters of two-layer LSTM predictor.

Parameters	Representations
σ	Sigmoid function
tanh	Hyperbolic tangent function
$i_{t}$	Input gate
$f_{t}$	Forgot gate
$o_{t}$	Output gate
$c_{t}$	State of current memory cell at time *t*
${\tilde{c}}_{t}$	Candidate value for state at time *t*
$h_{t}$	Output value
$x_{t}$	Input value
$W_{i}$, $w_{f}$, $w_{o}$, $w_{c}$	Weights
$R_{i}$, $R_{f}$, $R_{o}$, $R_{c}$	Weights
$b_{i}$, $b_{f}$, $b_{o}$, $b_{c}$	Bias vectors of three gates
∗	Element-wise multiplication

**Table 2:** Model parameters.

Parameters	Value
LSTM	2
Epoch	25
Batch size	32
Time step	10
Unit	50
Activation function	Relu
Optimizer	adam
Loss function	mean_squared_error

## Data Availability

NIST datasets are available at http://doi.org/10.18434/T44S3N, accessed on 1 January 2023.
